# Genomic spectrum of congenital heart disease combined with kidney and urinary tract anomalies uncovered by exome sequencing and array-CGH

**DOI:** 10.3389/fcell.2026.1862793

**Published:** 2026-09-09

**Authors:** Anna Zlotina, Sergei Zhuk, Ivan Kozyrev, Margarita Sorokina, Ekaterina Nikitina, Elena Shagimardanova, Olesia Melnik, Tatiana Vershinina, Natalia Petrova, Ilya Kagantsov, Elena Vasichkina, Tatiana Pervunina, Anna Kostareva

**Affiliations:** 1 Almazov National Medical Research Center, Saint Petersburg, Russia; 2 Life Improvement by Future Technologies (LIFT) Center, Moscow, Russia; 3 Loginov Moscow Clinical Scientific Center, Moscow, Russia; 4 Department of Women’s and Children’s Health, Karolinska Institutet, Stockholm, Sweden

**Keywords:** array-based comparative genomic hybridization, congenital anomalies of the kidney and urinary tract, cardiac mesenchymal cells, congenital heart disease, ROBO1, whole-exome sequencing

## Abstract

Congenital heart disease (CHD) combined with anomalies of the kidney and urinary tract (CAKUT) belongs to severe clinical conditions that represent isolated cases or can be part of complex inherited syndromes. To date, knowledge of the genetic basis of cardio-renal birth defects is very limited. Using chromosomal microarray analysis and exome sequencing, we investigated the spectrum of copy number variations (CNVs) and point genetic variants in a pediatric cohort of 30 patients presenting with combined CHD and CAKUT. Two patients possessed a pathogenic deletion in the 22q11.2 genomic region, well-known as the DiGeorge/velocardiofacial syndrome locus. Four other patients harbored rare copy number gains, which included one of the dosage-sensitive genes: *DHFR* (5q14.1), *NPHP1* (2q13), *NNT* (5p12), and *MCTP2* (15q26.2). In six children, novel or previously described causative variants were detected in the genes associated with orphan monogenic disorders such as Kabuki (*KMT2D)*, Noonan (*PTPN11*), Adams-Oliver (*NOTCH1*), and Alagille (*JAG1*) syndromes, as well as *SALL4*- and *MED13*-related syndromes. Furthermore, potentially disease-contributing variants were identified in the genes that primarily code for cardiac transcription factors, chromatin remodeling proteins, Notch- or *BMP-*signaling molecules, and components of cilia. Notably, two children had loss-of-function variants in *ROBO1*, encoding one of the receptors of the SLIT/ROBO signaling pathway. *In vitro* studies on cardiac mesenchymal cells demonstrated a dramatically diminished expression level of ROBO1 in a patient with novel compound heterozygous variants c.541_542del (p.Val181CysfsTer18) and c.657+4A>G and a moderately reduced ROBO1 expression in a patient with a heterozygous stop-gain variant c.2758C>T (p.Arg920Ter), which suggests a haploinsufficiency mechanism. Together, our findings underlined the utility of early cytogenetic screening for the 22q11.2 deletion among children with cardio-renal malformations and confirmed the importance of rare single-nucleotide variants in the genes involved in cardiogenesis and kidney development. In particular, *ROBO1* can be regarded as a candidate gene associated with combined heart and kidney structural defects and should be included in appropriate diagnostic gene-sequencing panels. The results support the usefulness of WES and array-based comparative genomic hybridization (array-CGH) analysis for new candidate gene identification, more accurate diagnosis, and family genetic counseling.

## 1 Introduction

Congenital heart disease (CHD) is the most frequently detected structural birth malformation (∼8/1000 newborns), still making a significant contribution to infant and child mortality and disability. CHD can occur as an isolated pathology, be combined with an extracardiac malformation, or be a part of complex syndromic phenotypes. In particular, the high rate of co-occurrence of CHDs and congenital anomalies of the kidney and urinary tract (CAKUT) has been noted. According to large-scale studies on the prevalence of such conditions, up to 34% of patients with CAKUT also have CHD (Greenwood et al., 1976; Cocchi et al., 1996; Adhisivam et al., 2005; Atić, 2016; Alp et al., 2022). Conversely, as much as 30% of children with cardiac malformations may present with a renal birth defect (Miller et al., 2011; Lowry et al., 2013; Egbe et al., 2014; San Agustin et al., 2016; Jiang et al., 2020). In addition, co-presence of cardiac and urogenital anomalies is also typical for some chromosomal and monogenic conditions, including Down syndrome, DiGeorge and Alagille syndromes, and VACTERL association. These notions suggest a genetic link between the two groups of structural anomalies and imply possible common molecular and cellular mechanisms of their formation during embryonic development (San Agustin et al., 2016; Barakat and Butler, 2024).

Over the past 2 decades, great progress has been made in understanding the genetic basis of different CHD types. Since high-throughput sequencing technologies were widely adopted in clinical and biomedical research, hundreds of CHD-related causative genes and molecular pathways have been identified (Geddes and Earing, 2018; Pierpont et al., 2018; Hill et al., 2022; Morton et al., 2022; Dong et al., 2025). The genetic background of renal birth defects is less understood; however, some of the principal CAKUT-related molecular determinants have been defined (Majumdar et al., 2003; Madariaga et al., 2013; Sanna-Cherchi et al., 2013; Hwang et al., 2014; Saygili et al., 2020). At the same time, knowledge of the genetic basis of cardio-renal birth defects is still limited to single clinical case reports and studies on mutant animal models (San Agustin et al., 2016).

In the present study, an integrative approach of exome sequencing and chromosomal microarray (CMA) analysis was applied to a cohort of 30 children born with severe CHDs and CAKUT. As a result, a spectrum of rare and novel causative or potentially disease-contributing variants was delineated in genes involved in different cellular processes and essential for cardiac and kidney development. In addition, a primary culture approach using patient-specific cardiac mesenchymal cells (CMCs) was employed as a relevant and informative model to determine the impact of genetic variants of uncertain clinical significance on the pathogenesis of congenital malformation.

## 2 Materials and methods

### 2.1 Cohort of patients

The study was performed in accordance with the Declaration of Helsinki and approved by the Ethical Committee of the Almazov National Medical Research Center. Written informed consent was obtained from the representatives of all patients.

The cohort included 30 pediatric patients aged 0-17 years (CHD/KD-#01- CHD/KD-#30) that were hospitalized to Almazov National Medical Research Center with a diagnosis of congenital heart disease and kidney/urinary tract malformation. We aimed to select more complex and severe cardiac and renal conditions where a stronger genetic background was suspected. The patients’ cardiac structural defects, CAKUT phenotypes, and co-malformations, if any, are listed in Supplementary Table S1. The cardiac abnormalities predominantly included ventricular septal defects (VSDs), tetralogy of Fallot (ToF), pulmonary valve stenosis, left heart hypoplasia syndrome (HLHS), atrioventricular canal, patent ductus arteriosus (PDA), and transposition of the great arteries (TGAs). As kidney malformation, renal agenesis/hypoplasia, duplex kidney, dystopia, «horseshoe»/L-shaped kidney, megaureter, and hydronephrosis were preferentially enrolled. Several exclusion criteria were applied. First, prior to enrollment, aneuploidies or large-scale structural chromosome abnormalities were excluded by standard karyotyping of peripheral blood lymphocytes. Second, we excluded cases of multiple congenital malformations and obvious monogenic syndromes suspected according to clinical signs and, then, genetically confirmed. Such conditions mainly included complete DiGeorge syndrome (cytogenetically verified), typical forms of Alagille/CHARGE/Noonan syndromes with multi-organ involvement, and VACTERL association. Finally, patients with severe neurological symptoms, global psychomotor or developmental delay, and psychiatric diagnoses (autism, severe epileptic seizures, etc.) were not enrolled. All the patients underwent appropriate cardiac and urologic surgical correction, and therapeutic treatment in the Almazov Center. For comprehensive genetic testing, peripheral blood samples from the probands were obtained. Parental blood samples were recruited where available.

### 2.2 Chromosome microarray analysis

Array-based comparative genomic hybridization (array-CGH) was performed using the SurePrint G3 Human CGH Microarray 8 × 60K with a median probe spacing of 41 kb (Agilent Technologies, United States). Genomic coordinates of probes refer to the human genome reference assembly NCBI37 (hg19). Genomic DNA was extracted from peripheral blood cells using FlexiGene DNA Kit (QIAGEN, United States). Commercial female and male DNA (Agilent Technologies, United States) was used as a reference in sex-matched hybridization. A measure of 200 ng of input sample and reference DNA was differentially labeled with Cy3 and Cy5, respectively. Hybridization, washing, and scanning procedures were carried out according to the manufacturer’s recommendations (Agilent Technologies). The data obtained were processed and analyzed using CytoGenomics software (v3.0.1.1). Copy number variants (CNVs: gains and losses) were called using an aberration detection statistical algorithm, ADM-2, with a sensitivity threshold of 6.0, a window size of 2 Kb, and a minimum number of probes for gain and loss ≥ 3. The revealed CNVs were evaluated based on data from publicly available databases of normal and pathogenic human genome variants, including the Database of Genomic Variants (DGV), Online Mendelian Inheritance in Man database (OMIM), DECIPHER, ClinVar, and PubMed.

### 2.3 Next-generation sequencing and Sanger sequencing

Whole-exome sequencing (WES) DNA libraries were prepared using the SureSelectXT Human All Exon target enrichment kit (Agilent Technologies, CA), pooled together, and analyzed using the Illumina HiSeq 2500 and NextSeq platforms (Illumina; San Diego, CA). One of the thirty patients (CHD/KD-#11) was subjected to sequencing of 206 genes associated with CHD, CAKUT, and ciliopathy phenotypes using the custom SureSelectXT targeted panel (Zlotina et al., 2024). Target sequencing data were processed as previously described (Zlotina et al., 2024).

In brief, the raw data were processed using the bioinformatics tools BWA-MEM, Picard, GATK, and Annovar. Reads were aligned to the GRCh38 version of the human genome assembly. Quality control parameters were as follows: Q30 proportion, ∼89.5%; Q20, ∼ 94.6%; and mean depth coverage across all probed regions, 119.22. Variant calling was performed using HaplotypeCaller v4.3.0.0 and DeepVariant v1.4.0. The structural and functional effects of identified genetic variants were predicted using SIFT, FATHMM, PolyPhen, MutationTaster, and CADD (a CADD-score ≥20 was considered for further analysis). Identified variants were filtered and annotated with Variant Effect Predictor (VEP) v104, incorporating population allele frequencies from the gnomAD and ExAC databases. Prior to manual curation, variants were selected on the basis of the following criteria: coverage depth >5 for SNPs and >10 for indels, AF in gnomAD <0.01, non-benign classification in ClinVar, protein sequence change, or splicing disturbance. All variants of interest were classified according to American College of Medical Genetics and Genomics (ACMG) recommendations (Richards et al., 2015).

For NGS data validation and targeted genetic testing of the probands’ parents (where available), Sanger sequencing was carried out using the BigDye Terminator Sequencing Kit and Genetic Analyzer AB3100 (Applied Biosystems/Hitachi, Japan). Gene-specific primers were designed using the NCBI Primer Blast tool (https://www.ncbi.nlm.nih.gov/tools/primer-blast/) (available upon request). The disease-associated variants were deposited in the ClinVar database (https://www.ncbi.nlm.nih.gov/clinvar/), accession numbers SCV007541366–SCV007541383.

### 2.4 Cardiac mesenchymal cell primary cultures

Cardiac mesenchymal cells (CMCs) were obtained from the patients’ myocardial tissue, propagated, and phenotyped, as previously described (Kozyrev et al., 2020). In brief, CMCs were isolated from cardiac tissue samples of the CHD/KD-#21 and CHD/KD-#22 patients and of five age-matched patients (1.5–6 years old) with an isolated subtle atrial septal defect (ASD) (as a comparison group) by mechanical dissection into tiny pieces and subsequent digestion by a 2 mg/mL collagenase II solution (Worthington) for 1.5 h in an incubator at 37 °C and 5% CO_2_. The cell suspensions were centrifuged at 300 *g* for 5 min; after that, the cell pellets were resuspended in growth medium (20% endothelial cell medium (Invitrogen, United States), 70% DMEM/F12 (Gibco Invitrogen, Waltham, MA, United States), 10% fetal bovine serum (HyClone, Logan, UT, United States), 100 μM MEM NEAA amino acid solution (Gibco, Grand Island, New York, United States), 2 mm L-Glutamine (Gibco, United States), and a mixture of penicillin (100 μ/mL) and streptomycin (100 μg/mL) (Gibco, United States)), seeded on the 25-cm^2^ flasks (Corning), and cultured at 37 °C and 5% CO_2_. At the third passage, the cells were re-seeded on 6-well plates (Corning) for subsequent RNA and protein extraction.

### 2.5 Reverse transcription-polymerase chain reaction

Total RNA was extracted from CMCs using ExtractRNA reagent (Evrogen, Russia). Approximately 1 μg of RNA was subjected to reverse transcription using the Moloney murine leukemia virus (MMLV) RT kit and a random primer according to the manufacturer’s recommendations (Evrogen, Russia); subsequently, cDNA samples were used as a template for real-time PCR. The reaction mixture also included 5X qPCR mix-HS SYBR + LowROX (Evrogen, Russia), gene-specific forward and reverse primers (10 μM each), and nuclease-free water. The primers designed using the NCBI Primer Blast tool were as follows: ROBO1_F 5′-GGA​GAG​AAG​GGA​GTC​AGA​ATC​TA-3′, ROBO1_R 5′-GTA​ATT​GTG​AGG​TCG​CCA​GTC-3`; GAPDH_F 5′-CAA​GGT​CAT​CCA​TGA​CAA​CTT​TG-3′, 5′-GTC​CAC​CAC​CCT​GTT​GCT​GTA​G-3`. Data analysis was carried out using the ΔΔCT method; the GAPDH housekeeping gene was used for normalization. The data were visualized using GraphPad Prism 8 software. The significance of differences between the groups was assessed using the unpaired non-parametric Mann–Whitney test (a *p*-value <0.05 was regarded as statistically significant).

To confirm that the c.657+4A>G variant disrupts splicing of ROBO1 pre-mRNA, a cDNA fragment corresponding to exons 4–8 of the ROBO1 transcript was amplified from the CHD/KD-#22 patient and control cDNA samples by standard PCR. Specific primers designed using the NCBI Primer-BLAST tool were as follows: ROBO1_cDNA_F 5′-ATT​ACC​TTG​GAG​AGG​CTG​TGA-3′ and ROBO1_cDNA_R 5′-GTG​TAT​GAA​CCC​ATG​TCA​CCA-3`. The size of the PCR products was then analyzed by electrophoresis in a 1.5% agarose gel, followed by Sanger sequencing (see above).

### 2.6 Immunofluorescence staining

CMCs were seeded on cover glass, fixed in 4% paraformaldehyde (PFA) for 15 min, and permeabilized with 0.5% Triton X-100 for 10 min at room temperature. Then, the cells were blocked with 15% FBS (Gibco) for 30 min, followed by incubation with primary antibodies against ROBO1 (HPA052968, produced in rabbit, Sigma, MO, United States) for 1.5 h and goat anti-rabbit Alexa Fluor 488-conjugated secondary antibodies (Molecular Probes, United States) for 45 min at room temperature. Cell nuclei were stained with 4′,6-diamidino-2-phenylindole (DAPI). The slides were evaluated using Zeiss Axio Observer Z1 and ZEN Blue software (Carl Zeiss Microscopy, Jena, Germany).

### 2.7 Western blotting

Total protein was extracted from CMC cultures using RIPA buffer supplemented with a protease inhibitor cocktail (Roche, United States). Cell lysates were incubated on ice for 30 min and subsequently clarified by centrifugation at 16,000 g. Next, protein samples were denatured by heating at 95 °C for 5 min in 4× Laemmli buffer containing β-mercaptoethanol. Protein separation was performed by SDS-PAGE in a 10% polyacrylamide gel (20 and 40 mA). The separated proteins were then transferred to a 0.45 μm nitrocellulose membrane (AppliChem, United States). After transfer, the membrane was blocked with 5% non-fat milk and incubated overnight at 4 °C with the primary antibodies against ROBO1 (HPA052968, Sigma) and beta-Actin (#A5316, Sigma) diluted at 1:1000. A secondary anti-mouse and anti-rabbit HRP-conjugated antibody (BioRad, United States) was used at a dilution of 1:20,000. Protein bands were visualized and quantified using the Fusion software. Then, the obtained bands were analyzed using the quantification analysis module of ImageJ (NIH, United States). The ROBO1 protein level was normalized to the corresponding beta-actin data for each sample.

## 3 Results

### 3.1 Rare copy number variants detected by chromosome microarray analysis

**FIGURE 1:**
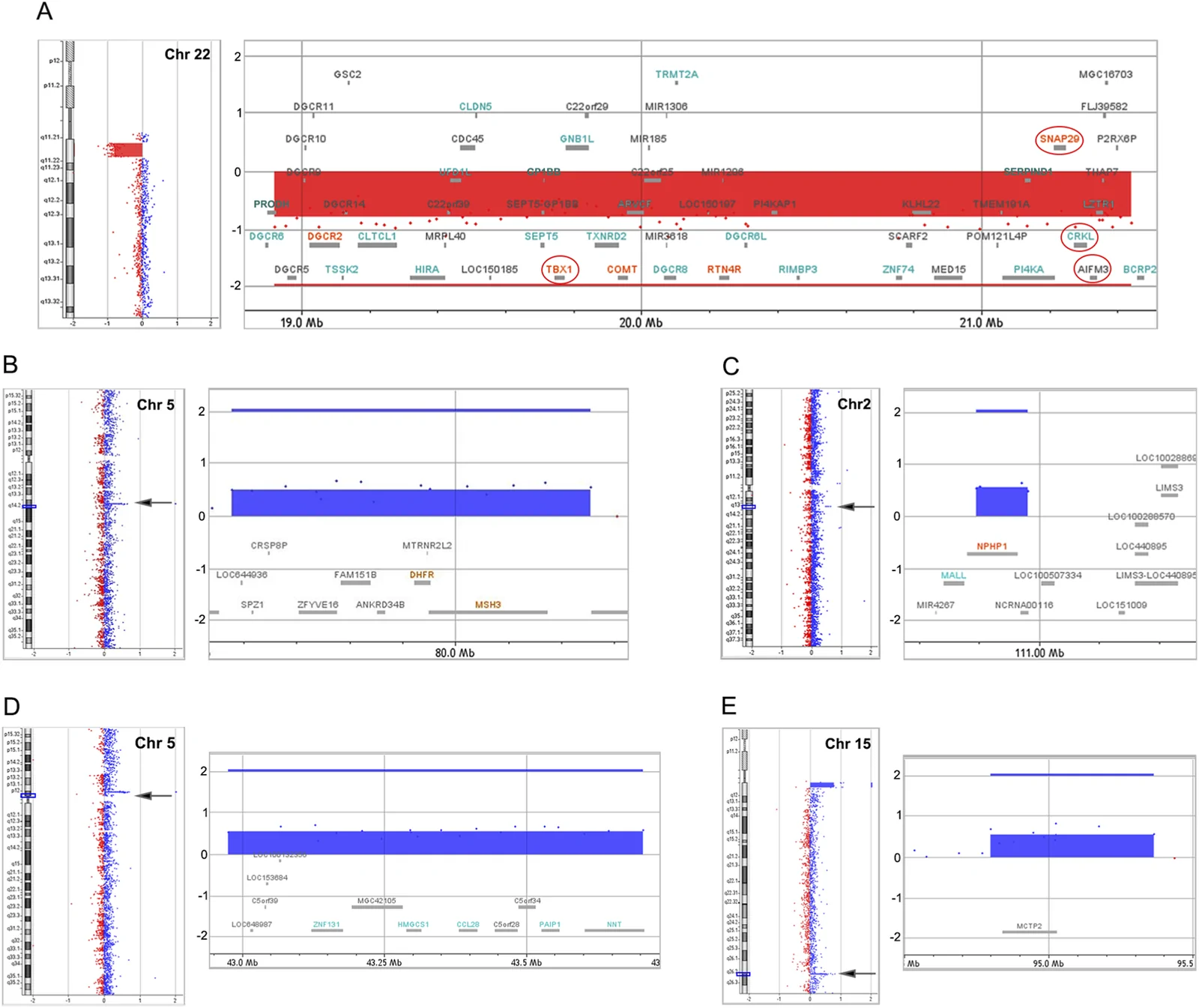
Detection of copy number variations using array-CGH. **(A)** Whole view of chromosome 22 (on the left) and the enlarged 22q11.2 region (on the right). Red rectangle indicates the deleted region. The putative causative genes are marked with a circle. **(B–E)** Whole view of chromosomes 5, 2, and 15 and enlarged 5q14.1, 2q13, 5p12, 15q26.2 loci. Blue rectangles indicate the duplicated regions. The microduplications are shown by an arrow.

Chromosome microarray analysis was performed to search for rare copy number variants–genomic gains and losses in the cohort. In two children (CHD/KD-#01 and CHD/KD-#02), a heterozygous 2.5-Mb deletion was identified in the 22q11.2 region known as the DiGeorge/velocardiofacial syndrome locus (Table 1; Figure 1A). No other pathogenic CNVs associated with well-established microdeletion or microduplication clinical syndromes were detected.

**TABLE 1:** Pathogenic and potentially disease-contributing CNVs identified by array-CGH.

Patient ID	Copy number variants (losses/gains)							
	Region	Size	Genomic coordinates, ISCN 2024	Candidate gene(s)	GnomAD SVs v2.1	Overlapping with known disease loci	Inheritance/genotype	DECIPHER/OMIM
CHD/KD#01	del 22q11.2	2.5 Mb	arr[GRCh37] 22q11.21(18,919,942_21440514)×1	*TBX1, CRKL*	f = 0.0001383	22q11 del syndrome (velocardiofacial/DiGeorge syndrome)	Unknown/het	P
CHD/KD#02	del 22q11.2	2.5 Mb	arr[GRCh37] 22q11.21(18,919,942_21440514)×1	*TBX1, CRKL*	f = 0.0001383	22q11 del syndrome (velocardiofacial/DiGeorge syndrome)	Unknown/het	P
CHD/KD#03	dup 5q14.1	680 kb	arr[GRCh37] 5q14.1(79,578,155_80254203)×3	*DHFR*	f = 0.00004610	N/A	Unknown/het	VUS
CHD/KD#04	dup 2q13	84 kb	arr[GRCh37] 2q13(110,895,981_110980401)×3	*NPHP1*	f = 0.0002305	N/A	Unknown/het	VUS
CHD/KD#05	dup 5p12	730 kb	arr[GRCh37] 5p12(42,975,303_43704937)×3	*NNT*	N/A	N/A	Unknown/het	VUS
CHD/KD#06	dup15q26.2	570 kb	arr[GRCh37] 15q26.2(94,798,428_95365520)×3	*MCTP2*	f = 0.00004610	N/A	Unknown/het	VUS

Four individuals possessed rare copy number gains of uncertain clinical significance (VUS) (Table 1). These duplications do not belong to common population variations: CNVs involving the same loci or overlapping genomic regions are few in number or absent in the GnomAD and DGV databases that collect variants distributed in healthy populations. The identified gains encompassed at least one clinically relevant and functionally important gene. In particular, the patient CHD/KD-#03 harbored a microduplication of ∼680 kb in size at the 5q14.1 locus (Figure 1B). Among several annotated genes involved in the duplicated region, there was an evolutionarily conserved *DHFR* encoding dihydrofolate reductase—a key enzyme in folate-mediated metabolism. The boy CHD/KD-#04 possessed a subtle microduplication at the 2q13 region (84 kb) (Figure 1C). The region comprises only two annotated genes, one of which is *NPHP1*, encoding nephrocystin-1, a ciliary transition zone protein. In patient CHD/KD-#05, a microduplication with a length of 730 kb was revealed at the 5p12 locus (Figure 1D). The duplication contained approximately 10 annotated genes, including *NNT*, which codes for nicotinamide nucleotide transhydrogenase, an integral protein of the mitochondrial inner membrane and one of the components of the respiratory chain. Finally, the girl with CHD/KD-#06 harbored a ∼570-kb microduplication at the 15q26.2 region (Figure 1E). The only protein-coding gene encompassed by the aberration is *MCTP2*, the product of which is an evolutionarily conserved protein with calcium ion-binding activity. The remaining children did not show any imbalances potentially contributing to their clinical phenotypes.

### 3.2 Disease-causing genetic variants uncovered by whole-exome sequencing

All children, except for two carriers of the 22q11.2 deletion, were further screened for single-nucleotide variants (SNVs) or indels using WES. Based on data analysis and further Sanger sequencing verification, a spectrum of pathogenic/likely pathogenic variants was identified in well-known disease-causing genes. In addition, variants of uncertain clinical significance were noted in relevant but poorly described genes or potential new causative candidates (Table 2).

**TABLE 2:** Pathogenic and potentially causative genetic variants uncovered by whole-exome sequencing.

Patient ID	Gene	Exon	Chrom. position (GRCh38)	Genetic variant (HGVS)	rs/ClinVar ID	GnomAD v4.1.0	CADD/SIFT/PolyPhen-2/ClinPred	Inheritance	ACMG classification
CHD/KD-#04	*NOTCH1*	Ex10	chr9:136,516,000	NM_017617.5:c.1649dup, p.Tyr550Ter	rs864622059	N/A	25.5/-/-/-	Unknown	P (PVS1, PM2, PM6, PP5)
CHD/KD-#07	*KMT2D*	Ex32	chr12:49,040,562	NM_003482.4:c.7207dup, p.Leu2403ProfsTer31	N/A	N/A	23.8/-/-/-	Unknown	LP (PVS1, PM2, PM6)
CHD/KD-#08	*PTPN11*	Ex3	chr12:112,450,358	NM_002834.5:c.178G>T, p.Gly60Cys	rs397507507	f = 6.199e-7	29.8/deleterious(0)/probably_damaging(0.999)/D	Unknown	P (PM2, PM5, PP3, PP5)
CHD/KD-#09	*SALL4*	Ex2	chr20:51,791,165	NM_020436.5:c.1318del, p.Gln440SerfsTer26	N/A	N/A	39/-/-/-	Unknown	LP (PVS1, PM2, PM6)
CHD/KD-#10	*MED13*	Ex15	chr17:61,984,356	NM_005121.3:c.2703T>A, p.Tyr901Ter	N/A	N/A	35/-/-/-	Unknown	LP (PVS1, PM2, PM6, PP3)
CHD/KD-#11	*JAG1*	Ex5	chr20:10,656,428	NM_000214.3:c.725A>G, p.His242Arg	ID:2,898,020	N/A	27.5/tolerated(0.05)/probably_damaging(0.993)/D	*de novo*	LP (PM2, PS2, PP3)
CHD/KD-#12	*NKX2-5*	Ex2	chr5:173232705	NM_004387.4:c.839C>T, p.Pro280Leu	rs761596254	ƒ = 0.00003789	21.4/tolerated(0.24)/benign(0.003)/T	Unknown	VUS (PM1, PM2)
CHD/KD-#13	*GATA5*	Ex5	chr20:62465914	NM_080473.5:c.833G>A, p.Arg278Gln	rs1259806979	ƒ = 0.000008176	29.6/deleterious(0)/probably damaging(0.999)/D	Unknown	VUS (PM2, PP3)
CHD/KD-#14	*SMAD6*	Ex1	chr15:66703290	NM_005585.5:c.32G>C, p.Arg11Pro	N/A	N/A	32/deleterious(0)/probably_damaging(0.953)/D	Unknown	VUS (PM2, PP3)
CHD/KD-#05	*GDF6*	Ex2	chr8:96145506	NM_001001557.4:c.425C>G, p.Pro142Arg	rs764057334	0.000007510	29.4/deleterious(0.05)/possibly)damaging(0.889)/D	Unknown	VUS (PM2, PP3
CHD/KD-#15	*PRDM6*	Ex5	chr5:123159537	NM_001136239.4:c.1052G>A, p.Cys351Tyr	rs773739323	ƒ = 0.00003416	27.2/deleterious(0.05)/probably_damaging(0.998)/D	Unknown	VUS (PM2, PP3)
CHD/KD-#16	*BAZ2B*	Ex27	chr2:159373164	NM_013450.4:c.4094T>C, p.Leu1365Pro	N/A	N/A	29.6/deleterious(0)/probably_damaging(0.999)/D	Unknown	VUS (PM2, PP3)
CHD/KD-#17	*CHD5*	Ex33	chr1:6121165	NM_015557.3:c.4852C>T, p.Arg1618Ter	ID:1307836	N/A	39/-/-/-	Unknown	LP (PVS1, PM2, PM6, PP3)
CHD/KD-#18	*WDR62*	Ex21	chr19:36097075	NM_001083961.2:c.2516G>A, p.Arg839Gln	rs766924001	ƒ = 0.000008056	28.7/deleterious(0)/probably_damaging(0.973)/D	Unknown	VUS (PM2, PP3)
CHD/KD-#19	*NOTCH4*	-	chr6:32196151	NM_004557.4:c.5299-1G>A	rs752207666	ƒ = 0.00001383	34/-/-/-	Unknown	VUS (PVS1, PM2)
CHD/KD-#20	*DNAH9*	Ex20	chr17:11690410	NM_001372.4:c.4588G>A, p.Glu1530Lys	rs746737529	ƒ = 0.00002479	28.3/deleterious(0.03)/possibly_damaging(0.861)/T	Unknown	VUS (PM2, PP3)
	*DNAAF5*	Ex1	chr7:727225	NM_017802.4:c.505C>T, p.Arg169Cys	N/A	N/A	23.8/deleterious(0)/benign(0.424)/D	Unknown	VUS (PM2, PP3)
CHD/KD-#21	*ROBO1*	Ex19	chr3:78651786	NM_002941.4:c.2758C>T, p.Arg920Ter	N/A	N/A	37/-/-/-	Unknown	P (PVS1, PM2, PP3, PP5)
CHD/KD-#22	*ROBO1*	Ex5	chr3:78746858	NM_002941.4:c.541_542del, p.Val181CysfsTer18	N/A	N/A	26.3/-/-/-	mat	LP (PVS1, PM2, PP3)
	*ROBO1*	-	chr3:78746739	NM_002941.4:c.657+4A>G	N/A	N/A	24.5/-/-/-	pat/*de novo*?	LP (PVS1, PS3, PM2)

HGVS, Human Genome Variation Society nomenclature; N/A–not available; gnomAD, allele frequencies in the gnomAD database (V`ersion:4.1.0); gnomAD SVs, allele frequencies in the gnomAD Structural variants Database (Version:2.1); D–damaging, T–tolerated;

P, pathogenic; LP, likely pathogenic; VUS, variant of unknown significance; PVS1, PS3, PM2, PM6, PP3, PP5, ACMG criteria of variant classification.

#### 3.2.1 Variants in genes associated with rare genetic syndromes

Several patients proved to harbor previously undocumented or earlier reported causative variants in the genes underlying rare monogenic syndromes commonly characterized by multiorgan involvement and dysmorphia including cardiovascular and kidney structural defects. In particular, the newborn girl CHD/KD-#07 with HLHS, complex renal malformation, and craniofacial features possessed a novel heterozygous frameshift variant c.7207dup (p.Leu2403ProfsTer31) in *KMT2D.* The gene encodes a histone H3 lysine 4-specific methyltransferase and is known as the principal gene responsible for Kabuki syndrome (KS). The boy CHD/KD-#08, presenting with aortic and pulmonary valve stenosis (as his mother), cryptorchidism, hydronephrosis, and pelviectasis, had a monoallelic missense variant c.178G>T (p.Gly60Cys) in the *PTPN11* gene, the product of which is a member of the protein tyrosine phosphatase (PTP) family. Germline pathogenic variants in *PTPN11* represent a genetic cause of complex multisystem conditions—RASopathies, the most common of which is Noonan syndrome (NS). Previously, this variant and other amino acid changes at the same protein position had been classified as likely pathogenic or pathogenic variants in individuals with Noonan syndrome spectrum disorders.

The proband CHD/KD-#04, who carried the 2q13 copy number gain, also possessed a 1-bp duplication variant c.1649dup (p.Tyr550Ter) in the *NOTCH1* gene, encoding one of the four NOTCH signaling receptors. Pathogenic mutations in *NOTCH1* have been reported as a genetic basis of different types of CHDs and a multisystem disorder—Adams–Oliver Syndrome (AOS). The nonsense variant c.1649dup was classified twice as pathogenic in ClinVar and comprehensively characterized as a genetic cause of AOS in a 3-generation family (Southgate et al., 2015). The girl CHD/KD-#09 with AV-canal/ASD combined with an L-shaped kidney and choanal atresia harbored a novel frameshift variant c.1318del (p.Gln440SerfsTer26) in *SALL4*. The gene codes for a C2H2 zinc finger transcription factor of the SAL type (Kohlhase et al., 2002) and, if mutated, causes various syndromic conditions such as Duane-radial ray syndrome (DRRS, Okihiro syndrome), acro-renal-ocular syndrome (AROS), and *SALL4*-related Holt–Oram syndrome (HOS). The boy CHD/KD-#10, who demonstrated TAPVC and VSD together with unilateral kidney agenesis and vesicoureteral reflux, carried a previously undescribed stop-gain variant c.2703T>A (p.Tyr901Ter) in *MED13* (mediator complex subunit 13) causative for syndromic phenotypes combining intellectual disability and dysmorphisms (Snijders Blok et al., 2018). Finally, the boy CHD/KD-#11, who had ToF with pulmonary valve atresia, kidney agenesis, and megaureter, had a missense variant c.725A>G (p.His242Arg) in the *JAG1* gene encoding the Notch ligand Jagged-1. Pathogenic variants in *JAG1* have been shown to explain more than 90% of Alagille syndrome cases. The *de novo* status of the c.725A>G in the proband was determined by Sanger sequencing of both parents.

#### 3.2.2 Variants in genes associated with non-syndromic cardiac and renal birth defects

In a number of cases, rare heterozygous variants of uncertain significance were detected in the genes earlier reported in relation to isolated birth defects of the heart and kidney. In particular, the boy CHD/KD-#12 possessed a missense variant c.839C>T (p.Pro280Leu) in a key cardiac transcription factor gene *NKX2-5.* The c.839C>T variant is located in a hot spot of variations with a pathogenic or VUS status and has several ClinVar records in association with cardiovascular phenotypes. The girl CHD/KD-#13 harbored a missense variant c.833G>A (p.Arg278Gln), affecting another principal cardiac transcription factor, GATA5, and showing high deleterious scores based on multiple computational tools. The girl CHD/KD-#14 had a novel missense variant c.32G>C (p.Arg11Pro) in the SMAD family member 6 (*SMAD6*) gene, also predicted to be a damaging substitution according to *in silico* algorithms. The boy CHD/KD-#05, who carried the 5p12 copy number gain, demonstrated a missense variant c.425C>G (p.Pro142Arg) in the *GDF6* gene, which encodes growth differentiation factor 6 belonging to the BMP family of ligands.

Several patients carried rare variants in genes implicated in maintenance and alteration of chromatin architecture. In particular, the proband CHD/KD-#15 harbored a missense substitution c.1052G>A (p.Cys351Tyr) in *PRDM6*, encoding a histone modifier; the patient CHD/KD-#16 had a c.4094T>C (p.Leu1365Pro) variant in *BAZ2B*, coding for another chromatin-remodeling protein, and the patient CHD/KD-#17 had a nonsense variant c.4852C>T (p.Arg1618Ter) in *CHD5* encoding a member of the chromodomain helicase DNA-binding protein family.

Among the other findings, the girl CHD/KD-#18 showed a missense variant c.2516G>A (p.Arg839Gln) in the cardiac-specific WD repeat-containing protein 62 (*WDR62*) gene. The girl CHD/KD-#19 possessed an acceptor splice-site variant c.5299-1G>A in the *NOTCH4* gene, encoding one more receptor of the NOTCH signaling pathway. Finally, the boy CHD/KD-#20 combined rare missense variants in two ciliary genes: c.4588G>A (p.Glu1530Lys) in *DNAH9* (dynein axonemal heavy chain 9) and c.505C>T (p.Arg169Cys) in *DNAAF5* (dynein axonemal assembly factor 5).

#### 3.2.3 Two cases of loss-of-function variants in the *ROBO1* gene

**FIGURE 2:**
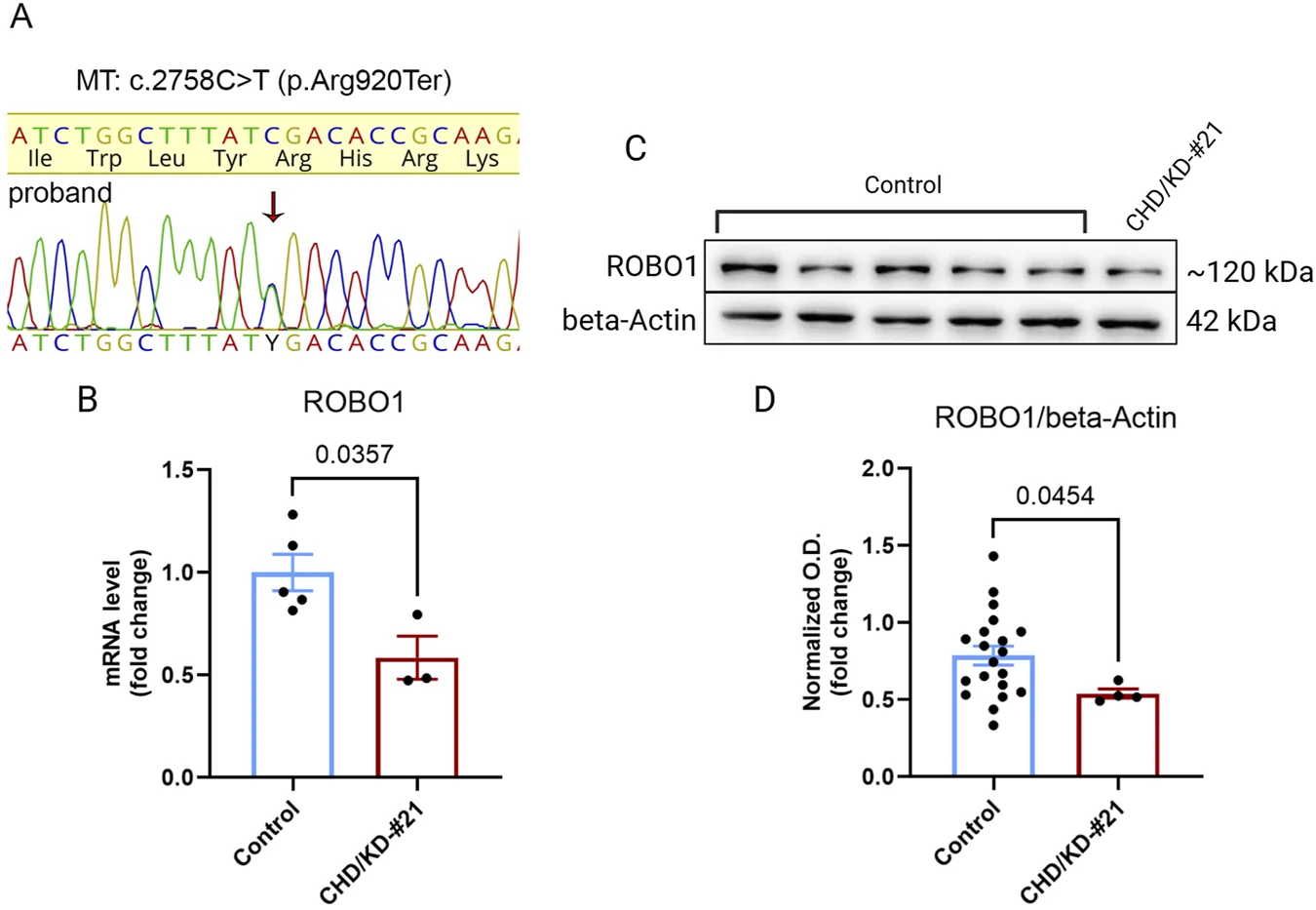
Characterization of the c.2758C>T (p.Arg920) variant in the *ROBO1* gene. **(A)** Verification of the c.2758C>T variant in the patient CHD/KD-#21 by Sanger sequencing. **(B)** Real-time PCR analysis of ROBO1 mRNA expression in CMCs of the proband (n = 3, technical replicates) relative to control cell cultures (n = 5, biological replicates); *p* = 0.0357. **(C,D)** Western blotting analysis of ROBO1 protein expression in CMCs of the proband (n = 3, technical replicates) relative to control cell cultures (n = 5 biological replicates, n = 15 technical replicates); fold change of ROBO1 protein expression in the patient relative to the control samples was quantified by densitometric scanning; *p* = 0.0454. Mann–Whitney test was used.

**FIGURE 3:**
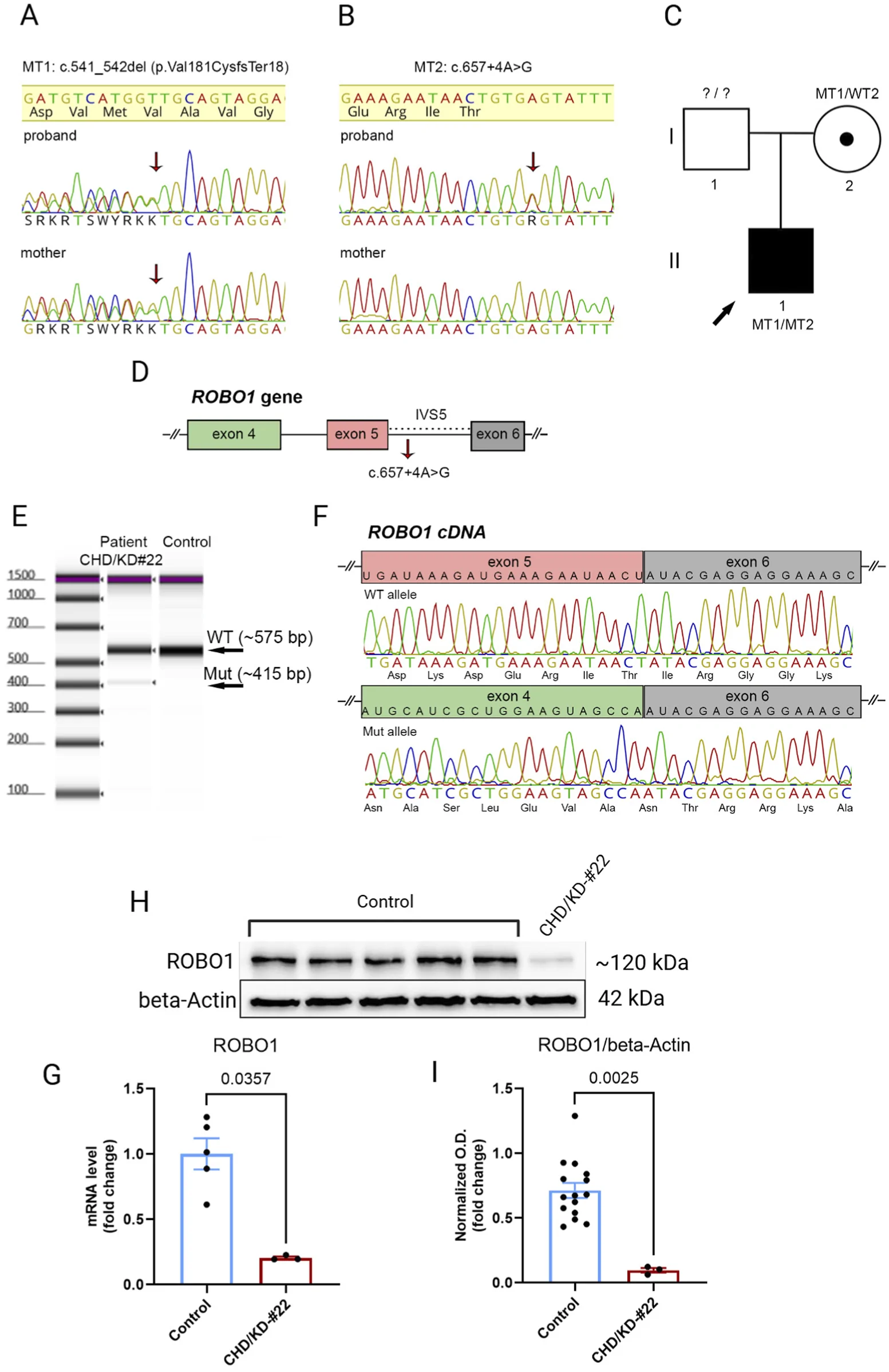
Characterization of the novel c.541_542del and c.657+4A>G variants in the ROBO1 gene. Verification of the c.541_542del variant, MT1 **(A)** and the c.657+4A>G variant, MT2 **(B)** in the patient CHD/KD-#22 by Sanger sequencing. **(C)** A family pedigree: the proband is a compound heterozygote for the c.541_542del (MT1) and c.657+4A>G (MT2) variants; the mother is a heterozygous carrier of MT1; the father is not genotyped. **(D-F)** Molecular characterization of the c.657+4A>G variant. **(D)** Schematic image of a *ROBO1* fragment comprising exons 4–6 with the c.657+4A>G variant pointed by an arrow. **(E)** Gel electrophoresis of RT-PCR samples from the patient and the control. **(F)** Sanger sequencing of RT-PCR products verified that a larger band (∼575 bp) corresponded to a WT allele (on the top), while a smaller band (∼415 bp) corresponded to a mutant allele lacking exon 5 (from below). **(G)** Real-time PCR analysis of ROBO1 mRNA expression in CMCs of the proband (n = 3, technical replicates) relative to control cell cultures (n = 5, biological replicates); *p* = 0.0357. **(H, I)** Western blotting analysis of ROBO1 protein expression in CMCs of the proband (n = 3, technical replicates) relative to control cell cultures (n = 5, biological replicates, n = 15 technical replicates); fold change of ROBO1 protein expression in the patient relative to the control samples was quantified by densitometric scanning; *p* = 0.0025. Mann–Whitney test was used.

Remarkably, two out of thirty children harbored variants in *ROBO1* (roundabout guidance receptor 1), presumably leading to a premature protein truncation (Table 2; Figures 2, 3). That is, the boy CHD/KD-#21 with ToF, megaureter, ureterohydronephrosis, and right-sided duplex kidney possessed a stop-gain variant c.2758C>T (p.Arg920Ter) (Figure 2A). The variant has a very low allele frequency in the healthy population and two ClinVar records as a pathogenic/likely pathogenic variant associated with the CAKUT condition or neurooculorenal syndrome with autosomal recessive inheritance (ID: 1,077,090) (Münch et al., 2022). The origin of this variant could not be determined due to unavailability of the parental blood samples. In another boy CHD/KD-#22 with a complete AV canal and left kidney agenesis, the novel frameshift variant c.541_542del (p.Val181CysfsTer18) and splice_donor_region variant c.657+4A>G were detected (Figures 3A,B). Targeted Sanger sequencing of the maternal DNA indicated that c.541_542del was inherited from the mother, while c.657+4A>G might have been inherited from the father or originated *de novo* (Figures 3A–C).

To determine the functional impact of the intronic variant c.657+4A>G, mROBO1 transcripts from the CHD/KD-#22 CMCs were evaluated. Particularly, we analyzed a cDNA region comprising exon 5 and flanking sequences (Figures 3D–F). On an agarose gel, a control PCR sample appeared as one band of the expected size (∼575 bp), apparently corresponding to a wild-type (WT) allele. At the same time, the patient’s PCR-product demonstrated two bands: a major band of the same size as that in the control (∼575 bp) and a weak band of ∼415 bp, presumably corresponding to a mutant allele lacking the exon 5 sequence (Figure 3E). Sanger sequencing of the PCR products confirmed that a larger fragment corresponded to a WT allele, while a smaller one corresponded to a mutant allele comprising the fused exon 4 and 6 sequences (Figure 3F). These findings allowed us to conclude that the c.657+4A>G variant disrupts the donor splice site and leads to skipping of exon 5 (158 bp), which predicts a premature stop codon due to an out-of-frame deletion.

To specify the molecular mechanisms mediated by the *ROBO1* genetic variants, we evaluated intracellular distribution as well as mRNA and protein expression levels of ROBO1 in primary cultures of CMCs of the patients CHD/KD-#21 and CHD/KD-#22 compared to cells isolated from ASD patients and used as a control. Immunostaining with the protein-specific antibodies did not reveal significant differences in intracellular ROBO1 distribution between the patients’ and control cells (data not shown). At the same time, according to both real-time PCR and Western blot analysis, ROBO1 expression was significantly decreased in patients’ cells compared to control cells (Figures 2B–D, 3G–I).

#### 3.2.4 Genetically negative cases

In eight patients (CHD/KD-#23 - #30), array-CGH and WES analyses proved to be uninformative and did not allow us to detect any pathogenic/likely pathogenic variants or VUSs in relevant genes potentially explaining the clinical phenotypes. Notably, cardiac and kidney malformations in such genetically negative patients were no less severe than cardio-renal defects in the genetically positive patients described above (Supplementary Table S1). In addition, some of these patients (for example, CHD/KD-#30) had a family history of structural heart and kidney defects, which also strongly implies some genetic background. The genetically uncharacterized cases could be associated with yet unreported causative genes or with undetectable genomic alterations in known morbid genes including mosaic mutations, subtle CNVs, variations in non-coding regions or regulatory elements, deep intronic splice site variants, variations disturbing 3D-genome structure, or some epigenetic anomalies.

## 4 Discussion

### 4.1 Contribution of structural genomic variants

In the present study, we aimed to delineate the clinical and genetic spectrum of combined CHDs and CAKUTs in a cohort of pediatric patients using high-resolution cytogenomic analysis and whole-exome sequencing. The genetic diagnostic yield of CMA was not high and was comparable to published data on CNV burden in cohorts with isolated CHDs or isolated CAKUTs (Ehrlich and Prakash, 2022; Li et al., 2022; Durbin et al., 2023). Here, the only unambiguously pathogenic loss, a heterozygous 22q11.2 deletion, was identified in two individuals, and four different gains of unknown significance were identified in four cases. The 22q11.2 deletion syndrome (22q11.2DS) is the most frequent chromosome microdeletion disorder, with a worldwide prevalence estimated to be 1 in 3,800–7,000 (McDonald-McGinn et al., 1993). The 22q11.2 deletion underlies multisystem, clinically overlapping conditions such as DiGeorge syndrome and velocardiofacial syndromes, conotruncal anomaly face syndrome, and Opitz G/BBB and Cayler cardiofacial syndromes. Remarkably, cases of 22q11.2 deletion are characterized by marked phenotypic heterogeneity, which may reflect variability in the length of the deleted region, the co-occurrence of deleterious genetic variants at this locus on a normal chromosome 22 homolog, or the presence of additional genetic modifiers in the genome. Our two patients bearing the 22q11.2 deletion did not demonstrate full complex clinical picture typical for DiGeorge or velocardiofacial syndromes but only had cardiac and renal defects. According to different investigations, as much as 60%–80% of patients with the 22q11.2 deletion have cardiac defects, the most typical of which are ToF (as in case CHD/KD-#01), VSD, interrupted aortic arch, and truncus arteriosus (as in case CHD/KD-#02) (Goldmuntz, 2020; Cirillo et al., 2022). Genitourinary abnormalities are observed in approximately 16%–30% of 22q11.2DS cases, with the most common defects being hydronephrosis (as in case CHD/KD-#01), renal agenesis, dysplastic or cystic kidney, vesicoureteral reflux (as in case CHD/KD-#02), cryptorchidism, and hypospadias (McDonald-McGinn et al., 1993; Wu et al., 2002; Campbell et al., 2018; Cirillo et al., 2022).

The common 22q11.2 deletion (referred to as a 2.54-Mb deletion based on CMA designs) encompasses more than 50 annotated genes, including the most investigated gene, T-box transcription factor 1*.* According to human genetic testing and studies on mouse mutant models, *TBX1* haploinsufficiency is regarded as a major molecular mechanism responsible for the cardiovascular phenotype observed in 22q11.2DS (Merscher et al., 2001; Yagi et al., 2003). WES screening of patients with CAKUT and further functional studies on mutant zebrafish and murine embryos defined the *SNAP29*, *AIFM3*, and *CRKL* genes within a ∼370-kb critical region at the 22q11.2 locus as causative for the kidney phenotype, with haploinsufficiency of *CRKL* being a principal genetic driver (Lopez-Rivera et al., 2017).

Rare copy number gains detected in four other children with various cardio-renal defects also comprised the genes (*DHFR*, *NPHP1*, *NNT*, and *MCTP2*) playing vital roles in cell homeostasis during embryonic development and postnatally, and regarded as dosage-sensitive genes in the literature. In particular, the *DHFR* gene, involved in folate metabolism, is indispensable for proper cardiogenesis. *DHFR* knockdown was shown to lead to malformations of the heart and outflow tract in zebrafish embryos, which could be due to its negative impact on the expression of key cardiac transcription factors *MEF2C, NKX2-5, TBX1*, and *TBX20*; cell proliferation; and apoptosis (Sun et al., 2011). Furthermore, previous array-CGH screening of patients with pulmonary atresia described a 5q14.1 microduplication, comprising *DHFR*, as a potential genetic basis of CHD (Xie et al., 2014). Consistent with this, in a recent study, a *dhfr* knock-in model of zebrafish, which resulted in DHFR overexpression, demonstrated severe cardiac defects and some gonadal abnormalities (Gong et al., 2021). Here, we, for the first time, describe a *DHFR* duplication in association with ventricular septal defect and hydronephrosis and, thus, expand the range of structural birth defects presumably caused by CNVs at this locus.

Deletions and loss-of-function (LoF) mutations of the *NPHP1* gene (2q13), mediating control of cell division and adhesion signaling pathways, underlie some ciliopathic phenotypes with renal anomalies, including juvenile nephronophthisis, Joubert, Senior–Loken, and Bardet–Biedl syndromes, and CHD cases (Riley et al., 2015). At the same time, recurrent 2q13 duplications of various sizes encompassing *NPHP1* have been previously reported in association with autism spectrum disorder, intellectual disability, liver disorders, dysmorphic features, and sometimes observed in a healthy control population (Baris et al., 2006; Riley et al., 2015; Chen et al., 2017). A case of intrachromosomal triplication of bands 2q12.3 to 2q13 was described in a patient with a polycystic kidney, PDA, abnormal uterus, and deafness (Mercer et al., 2009). The clinical implications of CNVs encompassing the nicotinamide nucleotide transhydrogenase (*NNT)* gene are not sufficiently clear. Homozygous and compound heterozygous *NNT* mutations lead to glucocorticoid deficiency (Meimaridou et al., 2012; Roucher-Boulez et al., 2016). Heterozygous LoF variants in *NNT* can also cause left ventricular noncompaction (Bainbridge et al., 2015). However, nothing is currently known about the potential impact of *NNT* copy number changes on heart or kidney structural defects. Finally, the *MCTP2* gene (15q26.2) encodes the evolutionarily conserved protein with calcium ion binding activity. The findings from the study on morpholino knockdown and mRNA overexpression of *Mctp2* in *X. laevis* allow it to be regarded as a dosage-sensitive gene essential for proper left ventricular outflow tract development (Lalani et al., 2013). Moreover, heterozygous deletion, intragenic duplications, and missense variants in *MCTP2* have been detected in single clinical cases of aorta coarctation and hypoplastic left heart syndrome (Lalani et al., 2013). Notably, such phenotypic features are consistent with the cardiac left-sided lesion in our patient bearing a 15q26.2 microduplication.

Together, apart from the pathogenic 22q11.2 deletion, several rare copy number gains that encompass genes widely expressed in heart and kidney tissues and play an important role during embryogenesis were detected. Additional functional studies are required to determine with certainty the clinical significance of these microimbalances and their potential contribution to cardio-renal clinical phenotypes.

### 4.2 Contribution of single-nucleotide variants

High-throughput sequencing proved to be more informative in detecting potentially disease-associated variants in the cohort. Six patients harbored previously undocumented or earlier reported deleterious variants in the genes *KMT2D*, *PTPN11, JAG1*, *NOTCH1*, *SALL4*, and *MED13* underlying rare genetic syndromes. All these genes encode important members of multifunctional signaling pathways regulating principal molecular and cell events during embryogenesis. *De novo* heterozygous variants in *KMT2D* are responsible for up to 75% of Kabuki syndrome cases (Golden et al., 2023; Neumann and Karnik, 2026). As much as 70% of patients with KS demonstrate CHD of various types, with septal defects and left-sided obstructive lesions being the most prevalent (Neumann and Karnik, 2026). Renal and genitourinary malformations are also common and can be observed in at least 20%–40% of KS cases (Merdler-Rabinowicz et al., 2021; Neumann and Karnik, 2026). The most typical CAKUTs are hydronephrosis, renal dysplasia or ectopy, horseshoe kidney, urethral duplication, or duplication of the renal collecting system. This agrees with the complex cardiac and renal phenotypes of our patient CHD/KD-#07 with the novel frameshift variant c.7207dup in *KMT2D*. Pathogenic missense variants in *PTPN11* explain about half of the cases of Noonan syndrome. According to different estimations, 50%–80% of NS patients present with CHD, the most common of which is pulmonary valve stenosis. Renal abnormalities are less typical of NS but are detected in ∼10% of patients, with dilatation of the renal pelvis being the most prevalent defect. Both heart and kidney malformations of the patient CHD/KD-#08 carrying the pathogenic c.178G>T (p.Gly60Cys) variant in *PTPN11* fit the diagnosis of NS.

Monoallelic pathogenic variants in *JAG1* underlie the majority of Alagille syndrome cases or can be a cause of isolated cardiac disease, such as peripheral pulmonary stenosis, ToF, and septal defects (Spinner et al., 1993). Various types of CAKUTs are reported in approximately 40% of the patients with Alagille syndrome and more often include renal dysplasia, solitary kidney, renal cysts, ureteropelvic obstruction, or renal tubular acidosis (Spinner et al., 1993; Kamath et al., 2012). Identification of the rare *de novo* variant c.725A>G (p.His242Arg) in *JAG1*, together with the clinical picture, which included particular cardio-renal defects and some anomalies of formation and segmentation of vertebral bodies, allowed us to suggest Alagille syndrome in the patient CHD/KD-#11. The patient CHD/KD-#04, diagnosed with HLHS and kidney agenesis/duplex kidney, possessed the pathogenic nonsense variant c.1649dup (p.Tyr550Ter) in *NOTCH1*. Since the first report identifying *NOTCH1* truncating mutations as a genetic cause of congenital heart disease was published, numerous *NOTCH1* variants of different types have been described in CHDs with both left-sided and right-sided localizations and in complex Adams–Oliver syndrome cases (Garg et al., 2005; Stanley et al., 2024). The impact of *NOTCH1* variants on kidney development is poorly understood; however, occasional renal involvement has been reported in patients with AOS (Stanley et al., 2024).

*SALL4* LoF variants are known to be responsible for several phenotypically heterogeneous and overlapping syndromic conditions, including AROS, Okihiro syndrome, and HOS. As part of these syndromes, various renal abnormalities (ectopia, horseshoe kidney, unilateral renal agenesis, hypoplasia, or vesico-ureteral reflux), cardiac defects (septal defects, ToF, or conduction anomalies), and choanal atresia have been repeatedly reported (Kohlhase, 1993). At the same time, the most typical features of SALL4-related phenotypes, such as radial ray malformations and ophthalmological abnormalities, were not noticed in our patient CHD/KD-#09. *MED13*-associated syndrome cases had been shown to include CHDs such as subaortic stenosis, septal defects, or tricuspid insufficiency; however, renal malformations seemed not to be typical for such conditions (Snijders Blok et al., 2018; Zhou et al., 2021). Thus, while the clinical significance of the variants in *KMT2D*, *PTPN11*, *NOTCH1*, and *JAG1* is evident, further evaluations and follow-up of the patients are needed to specify the contribution of the *SALL4* and *MED13* truncating variants to cardio-renal phenotypes. Our results confirm that WES is a powerful approach for the early diagnosis of rare genetic syndromes comprising heart and renal malformations, especially in cases of atypical or mild forms when it is difficult to suspect the diagnosis.

In several patients, potentially deleterious variants were uncovered in genes sporadically reported in isolated CHDs or CAKUT conditions. The genes differ in functions and encode transcription factors, chromatin remodeling proteins, Notch- *or BMP-*signaling molecules, or components of cilia, including *NKX2-5*, *GATA5*, *SMAD6*, *WDR62*, *PRDM6*, *DNAH9/DNAAF5*, *NOTCH4*, *GDF6*, *BAZ2B*, and *CHD5*. Such genes have been shown to ensure correct cardiogenesis and kidney development based on clinical studies and *in vivo/in vitro* models.

*NKX2-5* and *GATA5* encode transcription factors well-known to be responsible for proper heart development. *NKX2-5* and *GATA5* mutations have been repeatedly reported in patients with septal defects, ToF, pulmonary atresia, bicuspid aortic valve (BAV), DORV, or atrioventricular conduction anomalies (Schott et al., 1998; Laforest and Nemer, 2011; Wei et al., 2013; Shi et al., 2014). SMAD6 represents an intracellular inhibitor of the BMP signaling pathway, and accordingly, *SMAD6* deficiency frequently causes congenital aortic valve defects, along with other cardiovascular malformations (Luyckx et al., 2022). *WDR62* has recently been suggested as a new susceptibility gene for CHDs, primarily for VSD and ToF cases (Hao et al., 2022). Based on experiments on zebrafish and mouse models with *Wdr62* deficiency, Hao et al. (2022) demonstrated inhibited proliferation of cardiomyocytes due to impaired spindle assembly and dysregulated cell cycle and Hippo signaling pathways. *PRDM6* codes for a histone modifier, acting as a transcriptional suppressor of contractile proteins in vascular smooth muscle cells, which ensures the proper regulation of ductus remodeling (Li et al., 2016). Missense mutations in *PRDM6* have been reported as a genetic cause of autosomal-dominant, non-syndromic, isolated PDA (Li et al., 2016), which is in accordance with our data. One patient with TGA and megaureter carried heterozygous missense variants in two ciliary genes, namely, *DNAH9* and *DNAAF5.* Pathogenic variants in these genes have been repeatedly reported in association with primary ciliary dyskinesia (PCD), laterality defects, or isolated CHD cases (Chen et al., 2022; Horani et al., 2023).

To the best of our knowledge, no pathogenic variants in the *NOTCH4* nucleotide sequence have been reported in association with cardiovascular or renal birth defects. At the same time, patients with ToF were recently shown to demonstrate hypermethylation of a CpG site in the *NOTCH4* promoter region and, as a result, decreased NOTCH4 expression (Zhu et al., 2020). Rare heterozygous variants in *GDF6* have recently been uncovered as a cause of renal malformations, which was confirmed by knockout, knockdown, and rescue experiments in *Xenopus laevis* and mouse models (Martens et al., 2020). Initially, *de novo* deletions, stop-gain variants, or missense variants in *BAZ2B* have been identified in a group of patients with autism spectrum disorder, developmental delay, or intellectual disability (Scott et al., 2020). However, the phenotypes of individuals with various LoF mutations in *BAZ2B* seem to be broader and can also include congenital heart defects, vision problems, and male genital anomalies (Sewani et al., 2024). Similarly, missense and protein-truncating variants in *CHD5* were described in association with an autosomal dominant form of intellectual disability and epilepsy (Parenti et al., 2021). To our knowledge, no clinically relevant point mutations in *CHD5* have been described in individuals with any type of birth heart defects or CAKUTs. However, cardiovascular malformations, cardiomyopathy, and, in rare cases, renal abnormalities were previously reported as a part of clinical phenotypes in cohorts of patients with 1p36 deletion syndrome (Zaveri et al., 2014; Jacquin et al., 2023).

Finally, two patients with cardio-renal defects possessed LoF variants in *ROBO1.* The gene encodes one of the single-pass transmembrane roundabout (Robo) receptors mediating cell signaling through the conserved Slit–Robo pathway. This cascade regulates axon guidance, cell adhesion, proliferation, migration, cytoskeletal dynamics, angiogenesis, and other principal processes. In mammals, three Slit ligands (Slit1-3) and four Robo receptors (Robo1-4) are widely expressed in various tissues and organs; in particular, they show distinct spatio-temporal expression patterns during heart development (Zhao and Mommersteeg, 2018; Huang et al., 2022; Münch et al., 2022). Over the past decade, a significant number of pathogenic variants in the SLIT and ROBO genes have been described in patients with congenital malformations, including diaphragmatic hernia, neurodevelopmental abnormalities, and various heart defects. The latter largely included ToF, septal defects, BAV, and coarctation of the aorta (Mommersteeg et al., 2015; Kruszka et al., 2017; Huang et al., 2022). Mouse mutants for *Slit2*, *Slit3*, *Robo1*, and *Robo2* demonstrated a similar spectrum of heart disease, namely, septal and valve defects (Zhao and Mommersteeg, 2018). SLIT–ROBO signaling is also known to be crucial for kidney development, with its disturbance being implicated in congenital renal anomalies. In particular, mice mutant for Slit2 and Robo2 demonstrated multiplex kidneys (Grieshammer et al., 2004). Similarly, WES of a cohort of patients with CAKUT revealed mutations in *SLIT2* and *SRGAP1* (a small GTPase-activating protein mediating SLIT2–ROBO2 signaling) in individuals with duplicated collecting systems, dysplastic kidneys, or renal agenesis (Hwang et al., 2015). Later, biallelic or monoallelic variants in *ROBO1* were defined as a genetic cause of severe CAKUT conditions such as unilateral or bilateral kidney agenesis or dysplasia, duplex collecting system, posterior urethral valve, and vesicoureteral reflux (Rasmussen et al., 2018; Münch et al., 2022). To our knowledge, the combined cardio-renal phenotypes associated with Robo1 mutations had not been previously reported in patient cohorts. However, in a large-scale study on mutant animals, VSDs combined with bilateral multiplex kidneys were demonstrated in Robo1^−/−^ and Slit2^−/−^ mice (San Agustin et al., 2016), which correlates with our findings.

Cardiac mesenchymal cells and hiPSC-derived cardiomyocytes have been shown to be helpful for *in vitro* heart disease modeling (Kitani et al., 2020; Kozyrev et al., 2020; Docshin et al., 2022; Cao et al., 2025). Particularly, such cell models could be used to specify the clinical significance and functional effect of novel unclassified genetic variants. Here, functional analysis of mRNA transcripts from patients’ CMCs enabled us to verify the deleterious impact of the intronic VUS c.657+4A>G on the splicing process, resulting in whole-exon skipping. Moreover, quantitative evaluation of ROBO1 mRNA and protein levels in CMCs allowed for revelation of a moderately reduced ROBO1 expression in the patient harboring a mono-allelic stop-gain c.2758C>T variant and detection of a dramatically diminished ROBO1 expression in the patient bearing the compound heterozygous variants c.541_542del and c.657+4A>G. This could point to a gene dosage effect and functional haploinsufficiency as a disease molecular mechanism. To decipher the exact molecular mechanisms and cell signaling pathways triggered by *ROBO1* mutations during cardiogenesis and kidney development, further comprehensive functional studies are needed.

In conclusion, our data imply that rare subtle CNVs do not appear to contribute significantly to the pathogenesis of combined structural heart and kidney malformations. However, the findings confirm the utility of early cytogenetic screening for the 22q11.2 deletion among children with phenotypes different from classic 22q11.2DS, namely, with non-syndromic structural defects in the cardiovascular and urinary systems. The causative or novel candidate genes identified by whole-exome sequencing are functionally diverse and cover multiple cell processes. To date, these genes have been predominantly described in association with cardiac formation, and their possible contribution to kidney development, and, hence, CAKUT pathogenesis is yet to be clarified. The present study is the first report on *ROBO1* LoF variants in a cohort of patients with combined CHDs and CAKUT, with *in vitro* functional validation. Our findings, together with the earlier reported data, imply that *ROBO1* can be regarded as a candidate gene associated with combined cardio-renal malformations and should be included in appropriate diagnostic gene sequencing panels. The findings support the usefulness of WES and aCGH analysis, along with the use of patient-specific mesenchymal cells, for new candidate gene identification, more accurate diagnosis and prognosis, and family genetic counseling.

## 5 Study limitation

Combined congenital heart and kidney defects seem to be a clinically and genetically heterogeneous group of disorders. The cohort size does not allow us to obtain a comprehensive picture of the genomic landscape in this complex condition. In this study, we limited ourselves to 30 patients and focused on the in-depth clinical and genomic analysis of this group. When discussing the genomic findings, we rather focus on the functional spectrum of the causative genes and novel candidates, but not on the variant detection yield and percentage of genetically positive cases. Further sampling is required to shed more light on the spectrum of pathogenic genomic variants underlying cardiac and renal malformations. For most variants, no segregation analysis was possible due to the unavailability of parental blood samples. Functional studies were not performed for all cases but were conducted for the key novel genetic findings—two patients with ROBO1 LoF variants. The study included a comprehensive genetic analysis using array-CGH and exome-sequencing; however, some genetic anomalies, such as variants in non-coding regions and regulatory elements, mosaic events, and epigenetic or 3D-genome structure alterations, cannot be excluded.

## Data Availability

The disease-associated variants were deposited in the ClinVar database (https://www.ncbi.nlm.nih.gov/clinvar/), accession numbers SCV007541366-SCV007541383.
